# A Logical Inference

**Published:** 1887-01

**Authors:** Fred. A. Bellamy

**Affiliations:** Streatham.


ARTICLE VII.

### A LOGICAL INFERENCE.

BY FRED. A. BELLAMY, STREATHAM.

A gentleman, aged about 40, of exceptionally fine physique, consulted me in the early part of 1885, complaining of suffering excruciating neuralgic paroxysms in the right facial and temporal regions at irregular intervals—once or oftener—during the day and night. He had visited two dentists (one of high repute) who had extracted two sound teeth—having, as they asserted, diagnosed exostosis, which proved upon extraction incorrect—and the operations caused no modification of the pain. Patient, who had prior to this been under the care of a general practitioner, and was still undergoing systemic treatment, at this stage consulted me—some three week subsequent to the last of the former operations. His anguish was temporarily relieved, as he assured me he could now localize the seat of pain in the right lower six-year molar. I carefully examined the tooth he indicated, as also all the others, but could not detect a trace of decay in any of them, and they all responded freely to the thermal test. He appeared so elated at being able himself to positively centralize his trouble in the said tooth that he insisted upon having it immediately removed, observing " he had already suffered enough in the interests of science, and now he would like to have a word in the matter himself."

Persisting, naturally enough, on this line of argument, I, though loth to do his bidding, extracted the condemned tooth under gas, but to my disappointment found it, on examination, perfect in every particular. He left apparently satisfied that, at least, he had had his own way, and I saw no more of him till about a year after, when he again called upon me to ascertain if there was a "fish-bone" stuck in his *left upper* six-year molar. I failed to discover any foreign matter in the tooth or its vicinity, but found I could introduce a fine probe in a crown fissure of the same tooth, and by my advice he returned the following day to have it stopped. Before commencing operations, I inquired whether the former neuralgic pains had disappeared. He told me that as the last extraction recorded above effected no benefit, he lost faith in dental assistance, and consulted first one, then another of our most eminent physicians and specialists; but no relief could they extend to him, till the last one he consulted required him to sever himself from London and business for a while and retreat to the mountains of Switzerland. This advice he followed as a *dernier ressort*, dwelling for a few weeks in a village situate at a great altitude, where the rarified and pure atmosphere invigorates the nerves to a healthy reaction. After a few days' sojourn he said the effect was marvellous; those awful paroxysms totally disappeared, he felt another man and "lived" again.

Contenting myself with the examination of the previous day, I immediately set to work to bur out the fissure, and after nearly impinging upon the pulp chamber without obliterating the crevice, I ceased excavating, suspecting the tooth might be cracked instead of attacked by caries—a suspicion that a closer examination confirmed—for I found the tooth split through its entire length, the two buccal roots being attached to the one half and the palatine to the other—the two halves being very slightly individually mobile. As a matter of course there was no vitality existing in the pulp, so I tightly bound the tooth around its neck with gold binding wire (the neck being too constricted to allow a collar to be slipped over the crown), and having further excavated the two halves after a dovetail fashion, inserted a filling of Sullivan's cement, and I believe the operation may be pronounced a success.

Now, would it be a very illogical hypothesis—no new one—to assume that that identical tooth was the origin of all the agony the patient had endured; the pulp having sphacelated, the gases generated by which found no exit, till the tension on the surrounding walls was greater than the natural cohesion of the molecules of the tooth-substance could resist, when they separated to allow the pressure to diffuse itself? I was informed by the patient that he had never used his teeth rashly, nor could he recollect any particular sensation at any time in connection with this tooth, save on the occasion when he imagined a fish-bone had become fixed in it. Might one not even go so far as to deduce from the history of the case, that his relief dated from the bursting of the tooth, which took place at the Swiss village, and which was possibly caused or accelerated by the altered climatic conditions surrounding it at that time?

There is, as I have stated, nothing novel in this—on the contrary, the theory here propounded is antiquated—

nor is there attached to either of the incidents here chronicled any special interest, if taken separately; but studied conjointly and in their sequence, it decidedly, I think, confirms the legitimate conclusions of yore, as to the power of the gas evolved from the disorganized pulp tissue within a tooth.—*London Dental Record.*

